# Baicalin Weakens *Staphylococcus aureus* Pathogenicity by Targeting Sortase B

**DOI:** 10.3389/fcimb.2018.00418

**Published:** 2018-11-30

**Authors:** Guizhen Wang, Yawen Gao, Hongsu Wang, Xiaodi Niu, Jianfeng Wang

**Affiliations:** ^1^College of Food Science and Engineering, Jilin University, Changchun, China; ^2^Key Laboratory of Zoonosis, Ministry of Education, College of Veterinary Medicine, Jilin University, Changchun, China

**Keywords:** *Staphylococcus aureus*, baicalin, sortase B, cytotoxicity, adhesion, inflammation, molecular dynamics simulation

## Abstract

*Staphylococcus aureus* (*S. aureus*) is a human and other animal pathogen that contributes to the primary etiology of nosocomial pneumonia, a disease with high mortality rates and costs. Treatment of multidrug-resistant *S. aureus* infection is extremely challenging, and new therapeutic strategies beyond antibiotic treatment are needed. Anti-virulence agents that specifically target the molecular determinants of virulence may be a novel method for treating drug-resistant nosocomial infections. Sortase B (SrtB) is a crucial virulence factor in *S. aureus* and plays an important role during infection. In this study, we find that baicalin suppresses the activity of SrtB. Minimum inhibitory concentration and growth curve assays confirmed that baicalin has no anti-*S. aureus* properties. We performed live/dead, lactate dehydrogenase (LDH), adherence, and enzyme-linked immunosorbent assays to confirm that baicalin reduced human alveolar epithelial A549 cell injury caused by *S. aureus*, reduced the adherence of *S. aureus* to A549 cells, and significantly attenuated the inflammatory response of mouse macrophage J774 cells to *S. aureus*. Additionally, we were able to elucidate the binding mechanics and identify the interacting sites of baicalin and SrtB via a molecular dynamics simulation, site-directed mutagenesis, and fluorescence spectroscopy quenching. Finally, we confirmed that baicalin directly binds to the active center of SrtB, and the residues Asn^92^ and Tyr^128^ perform an important function in the interaction of SrtB and baicalin. Taken together, these data indicate that baicalin is a promising candidate to combat *S. aureus* infections.

## Introduction

With the rapid emergence of antibiotic-resistant bacteria, new strategies aimed at targeting the virulence factors of pathogenic bacteria, rather than killing the bacteria directly, are needed to combat resistant bacterial infection (Moreillon, 2008; Benny, 2014; Mühlen and Dersch, 2016).

Drug-resistant strains of *S. aureus* have become some of the most prevalent pathogens of community-acquired and nosocomially acquired pneumonia, giving rise to high morbidity, mortality, and cost (Cascioferro et al., 2014). In particular, multidrug-resistant *S. aureus* strains have seriously affected human health and pose a great challenge to public health while imposing a substantial socioeconomic burden; as a result, this situation has attracted the attention of researchers worldwide (Schillaci et al., 2017). Therefore, exploring new drug targets to investigate corresponding inhibitors and validating the mechanism of action may result in new prospects and novel therapeutic agents.

SrtB is a transpeptidase of *S. aureus*; it anchors proteins to the cell wall by identifying specific sorting signals (Mazmanian et al., 2002; Zong et al., 2004; Bradshaw et al., 2015). IsdC is a substrate of SrtB and can be anchored to the cell wall by it. IsdC is an important rate-limiting protein for the process of capturing and transferring iron by *S. aureus* for successful infection (Maresso and Schneewind, 2006; Tiedemann et al., 2008; Villareal et al., 2011). During catalysis, a cysteine residue from the active site of SrtB and the cleaved substrate form an acyl intermediate, which is then resolved by the amino group of pentaglycine cross-bridges and is eventually anchored to the cytoderm (Marraffini and Schneewind, 2005).

Many studies have been conducted to elucidate the role of SrtB in the pathogenesis of *S. aureus* infection, and the results show that SrtB is crucial for *S. aureus* infections. Mazmanian et al. established a mouse model of arthritis involving *S. aureus* and its SrtB knockout mutant. The SrtB gene knockout group had lower survival rates, lower weight loss, attenuated inflammation, lower colony counts in joints and kidneys, lower clinical severity of arthritis, and much lower infection persistence (Jonsson et al., 2003). These observations suggest that SrtB is deeply involved in the development of murine arthritis. Subsequently, another report has described for the first time the active site disposition and the unique Cys-Arg catalytic machinery of SrtB on the basis of the crystal structures of SrtB in a complex with an active site inhibitor or the cell wall substrate analog tripleglycine (Zong et al., 2004). These studies formed the basis for further research on SrtB with novel small-molecule inhibitors of this enzyme. Additionally, Ki-Bong Oh et al. carried out a fibronectin-binding assay using *S. aureus* and its SrTB gene knockout strain, and found that adhesion to fibronectin was weakened in the SrTB gene knockout group compared to the wild-type group (WT) (Oh et al., 2005), confirming that SrtB is involved in the adhesion of *S. aureus* to host tissue. More recently, Jacobitz et al. revealed that the back-bone amide of Glu^224^ and the side chain of Arg^233^ form an oxyanion hole in SrtB to stabilize high energy tetrahedral catalytic intermediates. They also reported that a highly conserved Thr residue within the bound sorting signal substrate facilitates the formation of the oxyanion hole by stabilizing the position of the active site Arg residue via hydrogen bonding (Jacobitz et al., 2014). These data laid the foundation for studying the mechanism of interaction between SrtB and its inhibitors. These reports suggest that SrtB is a critical virulence factor of *S. aureus* and performs an important function in *S. aureus* infections.

According to current research, SrtB is crucial for *S. aureus* infections, suggesting that the development of inhibitors targeting SrtB may be a novel strategy to combat *S. aureus* infections. Elucidation of the interaction between SrtB and an inhibitor may also provide a theoretical rationale for treatments of *S. aureus* infections. Nonetheless, reports on SrtB inhibitors are scarce. In this study, we used SrtB as a target to screen the inhibitor and to determine the mechanism of interaction between SrtB and the inhibitor, aiming to provide useful information for better treatment of *S. aureus* infections.

Baicalin, a major bioactive component of Scutellaria which is a traditional Chinese medicine herb, has been demonstrated to possess multiple pharmacological activities, such as anti-inflammatory, anti-oxidant, and anti-tumor activities (Luan et al., 2018; Sherwani et al., 2018). In this study, we confirmed that baicalin has no anti-*S. aureus* properties but inhibits SrtB activity significantly. Live/dead and LDH assays indicated that baicalin protects human alveolar epithelial A549 cells from *S. aureus-*mediated cellular injury. Adhesion assays confirmed that baicalin weakens the adhesion of *S. aureus* to A549 cells. An enzyme-linked immunosorbent assay (ELISA) suggested that baicalin alleviates the inflammation-related activities of murine macrophagic J774 cells. Additionally, molecular dynamics simulations indicated that baicalin directly binds to the active center of SrtB and obstructs the interaction between SrtB and its substrate, causing a loss of SrtB activity. Site-directed mutagenesis and fluorescence spectroscopy quenching experiments confirmed that the residues Asn^92^ and Tyr^128^ are important for this process.

## Materials and methods

### Materials

Baicalin was purchased from Sigma-Aldrich (St. Louis, MO, USA). Fluorescent substrate peptide Dabcyl-QANPQTNEE-Edans was purchased from Shanghai GL Biochem Co., Ltd (GL Biochem, Shanghai, China).

### Expression and purification of SrtB_**Δ**30_ and its mutants

The DNA sequence of SrtB_Δ30_ was amplified from *S. aureus* 29213 genomic DNA as a template and was cloned into pET-28a. After that, the plasmid was transfected into *E. coli* BL21 (DE3) for expression.

The mutants of SrtB_Δ30_ (N92A, Y128A) were produced from the pET-28a plasmid using the Quick Change site-directed mutation kit (Stratagene, La Jolla, CA, USA). After digestion with the *Dpn*I enzyme, the plasmids were transfected into *E. coli* BL21 (DE3). The primer pairs used in this study are presented in Table S1.

*E. coli* was cultured in the LuriaBertani (LB) medium. Isopropyl-β-d-thiogalactoside (IPTG) was added when the optical density at 600 nm (OD_600_) reached 0.8. The bacteria were pelleted by centrifugation and lysed ultrasonically. The supernatant was loaded onto a His-affinity column (GE Healthcare Life Sciences). The contaminant proteins were eliminated with 10 mM imidazole in an equilibration buffer (300 mM NaCl, 50 mM NaH_2_PO_4_, 10 mM Tris-HCl, pH 8.0). SrtB_Δ30_ and the mutant proteins were eluted with 200 mM imidazole in an equilibration buffer.

### SrtB activity assay

Fluorescence resonance energy transfer (FRET) (Kang et al., 2006; Li et al., 2016) analysis was carried out to confirm the inhibitory effect of baicalin on SrtB. SrtB with various concentrations (0, 4, 8, 16, or 32 μg/ml) of baicalin in a buffer (50 mM Tris-HCl, 150 mM NaCl, pH 7.5) was incubated for 30 min at 37°C, followed by the addition of the fluorescent peptide substrate and incubation at 37°C for 1 h. The fluorescence values were recorded at 350 nm excitation wavelength and 520 nm emission wavelength. Proteinase K and an empty buffer served as the positive and negative controls, respectively.

### Susceptibility testing and growth dynamics assay

#### The susceptibility test

Minimum inhibitory concentrations (MICs) of baicalin for *S. aureus* strains were determined by the broth micro-dilution method according to the Clinical and Laboratory Standards Institute (CLSI) guidelines. Oxacillin served as a quality control.

#### Growth dynamics assay

*S. aureus* was cultured in the tryptic soy broth (TSB) medium until OD_600_ reached 0.3 and was then treated with various concentrations of baicalin (0, 8, 16, 32, or 64 μg/ml) and cultured at 37°C with shaking. OD_600_ was recorded every 30 min until the microbe reached the stationary phase.

#### Cytotoxicity and live/dead assays

Human alveolar epithelial A549 cells were cultivated in Dulbecco's modified Eagle's medium (DMEM), with 10% fetal bovine serum (Invitrogen). After dispersal with 0.25% trypsin, the cells were seeded in 96-well plates at 2 × 10^4^ per well and cultured at 37°C and 5% CO_2_ for 20 h. Next, fresh DMEM with *S. aureus* (the bacterial density was 4.2 × 10^7^ CFU/ml) and different concentrations of baicalin was used to replace the culture medium and was incubated for 5 h. The Cytotoxicity Assay Kit (LDH) (Roche, Basel, Switzerland) and the live/dead (green/red) reagent were employed to confirm cell viability according to the details described previously (Dong et al., 2013).

#### Adhesion test

A549 cells were seeded in 24-well plates at 2.5 × 10^5^ per well and incubated at 37°C with 5% CO_2_ for 20 h. *S. aureus* (the bacterial density was 2 × 10^7^ CFU/ml) with various concentrations of baicalin in DMEM was used to replace the culture medium and was incubated for 2 h. After that, the cells were dispersed with 0.25% trypsin and gently swirled in 0.2% Triton X-100. The colony count of *S. aureus* that adhered to A549 cells was determined by plate colony counting based on a method reported elsewhere (Song et al., 2017).

#### Enzyme-linked immune-sorbent assay (ELISA)

*S. aureus* pneumonia has become one of the most prevalent diseases in hospital- and community-acquired pneumonia. The inflammation caused by the *S. aureus* SrtB knockout strain was lower than that of wild type *S. aureus* (Jonsson et al., 2003), suggesting SrtB plays a role in the inflammation caused by *S. aureus*.

To explore the latent mitigating role of baicalin on the inflammation generated by *S. aureus*, an enzyme-linked immune-sorbent assay was carried out to determine the cytokine in the culture media of J774 mouse macrophage cells treated with *S. aureus*.

Mouse macrophage J774 cells were seeded in 24-well plates at 3 × 10^5^ per well and cultured at 37°C with 5% CO_2_ for 20 h. *S. aureus* (bacterial density was 1.2 × 10^6^ CFU/ml) with various concentrations of baicalin in DMEM was used to replace the culture medium with subsequent coculturing for 8 h. The supernatant was collected and cytokines (TNF-α, IL-1β, and IL-6) were quantified by an ELISA kit (Invitrogen, Thermo Fisher) according to the specifications. Cells treated with DMEM or *S. aureus* without baicalin served as the negative control and positive control, respectively.

### Molecular dynamics simulation

Molecular docking assay was carried out by means of the Auto Dock 4.0 software package in accordance with the crystal structure of SrtB (PDB code: 1NG5). The three-dimensional (3D) structure of baicalin was optimized in Gaussian 09 software at the B3LYP/6-311G^*^ level. The molecular dynamics simulation was performed on the structure of the SrtB-baicalin complex obtained by molecular docking. The detailed processes and methods are described in the Supplementary Materials.

### Statistical analysis

The data were analyzed in the SPSS 17.0 software by the independent Student's *t*-test. Results were considered statistically significant at *p* < 0.05.

## Results

### Baicalin inhibited the activity of SrtB

Previous reports have revealed that *S. aureus* SrtB can cleave sorting signal NPQTN. In the present study, the NPQTN oligopeptide with fluorescent groups at both ends served as the substrate of SrtB to evaluate the inhibitory effect of baicalin on SrtB.

**Figure 1:**
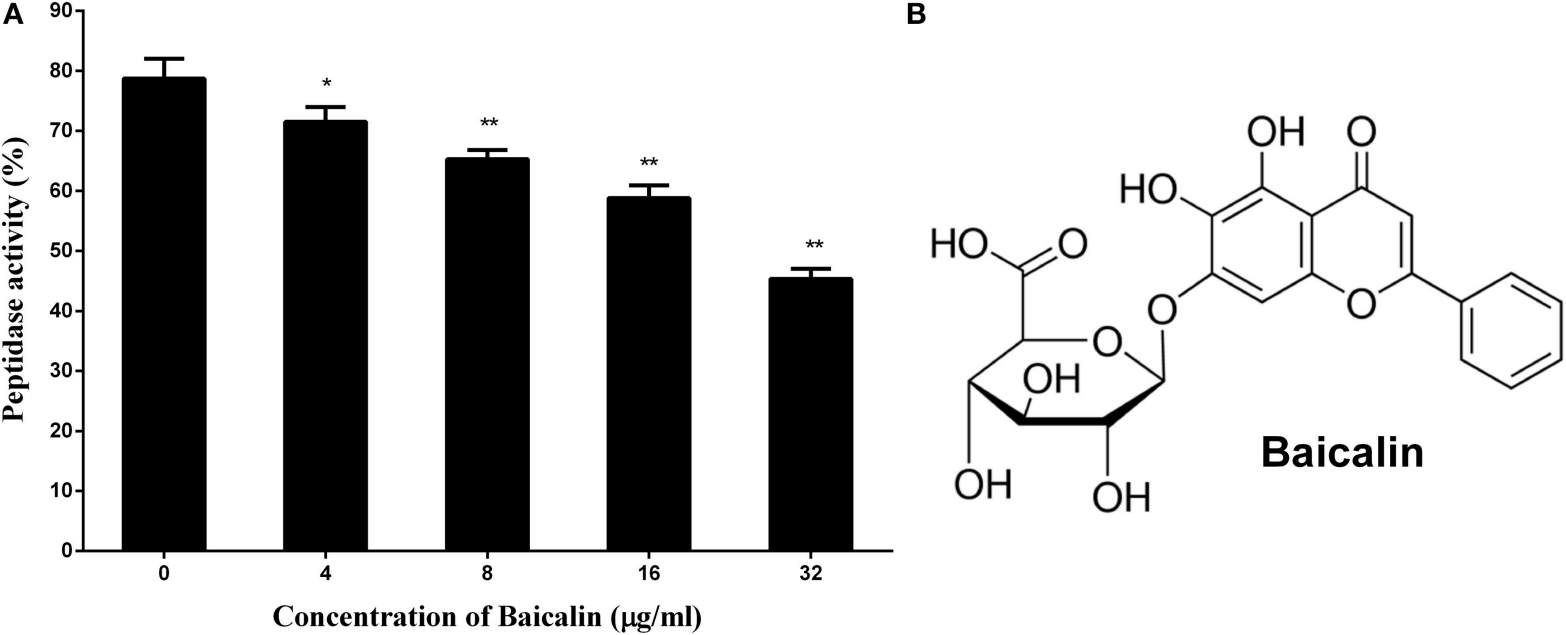
The inhibitory effect of baicalin on SrtB and the chemical structure of baicalin. **(A)** Baicalin inhibits the activity of SrtB. The data are shown as the mean values and standard deviation of three independent experiments. *p < 0.05, **p < 0.01. **(B)** The chemical structure of baicalin.

As depicted in Figure 1A, the activity of SrtB shows a gradual decrease in step with the increase in the concentration of baicalin, suggesting that baicalin inhibits SrtB activity in a dose-dependent manner. Figure 1B shows the chemical structure of baicalin.

### Baicalin exerted no growth pressure on *S. aureus* 29213

Growth pressure is the main cause of bacterial resistance to antibiotics. To test whether baicalin affects the growth of the tested strain, we conducted MIC and growth curve assays.

Baicalin did not exert growth pressure on *S. aureus* 29213 under the conditions of this assay, with the concentration of baicalin reaching 1,024 μg/ml as shown in Table 1.

**Table 1:** The minimal inhibitory concentrations (MICs) of baicalin against *S. aureus*.

**Inhibitors**	**Strains**			
	**29213**	**BAA-1717**	**BAA-1707**	**8325-4**
Baicalin (μg/ml)	>1,024	>1,024	>1,024	>1,024
Oxacillin (μg/ml)	0.25	128	64	0.125

**Figure 2:**
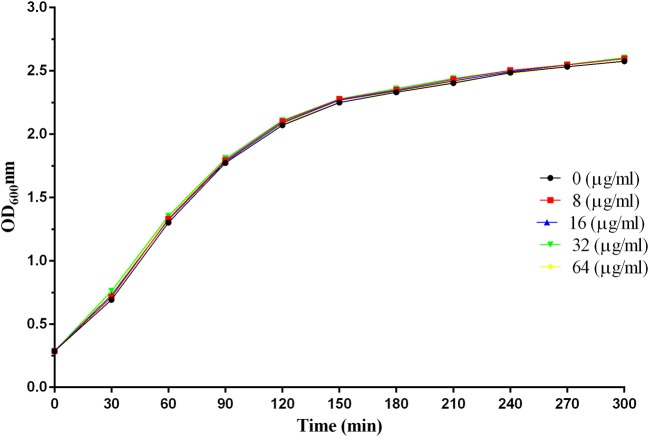
The growth curves of *S. aureus* 29213 at different concentrations of baicalin.

As illustrated in Figure 2, there were no significant differences in the growth trends of *S. aureus* with or without the addition of baicalin, indicating that baicalin does not affect the growth of *S. aureus*.

### Baicalin reduced human alveolar epithelial cell injury caused by *S. aureus*

**Figure 3:**
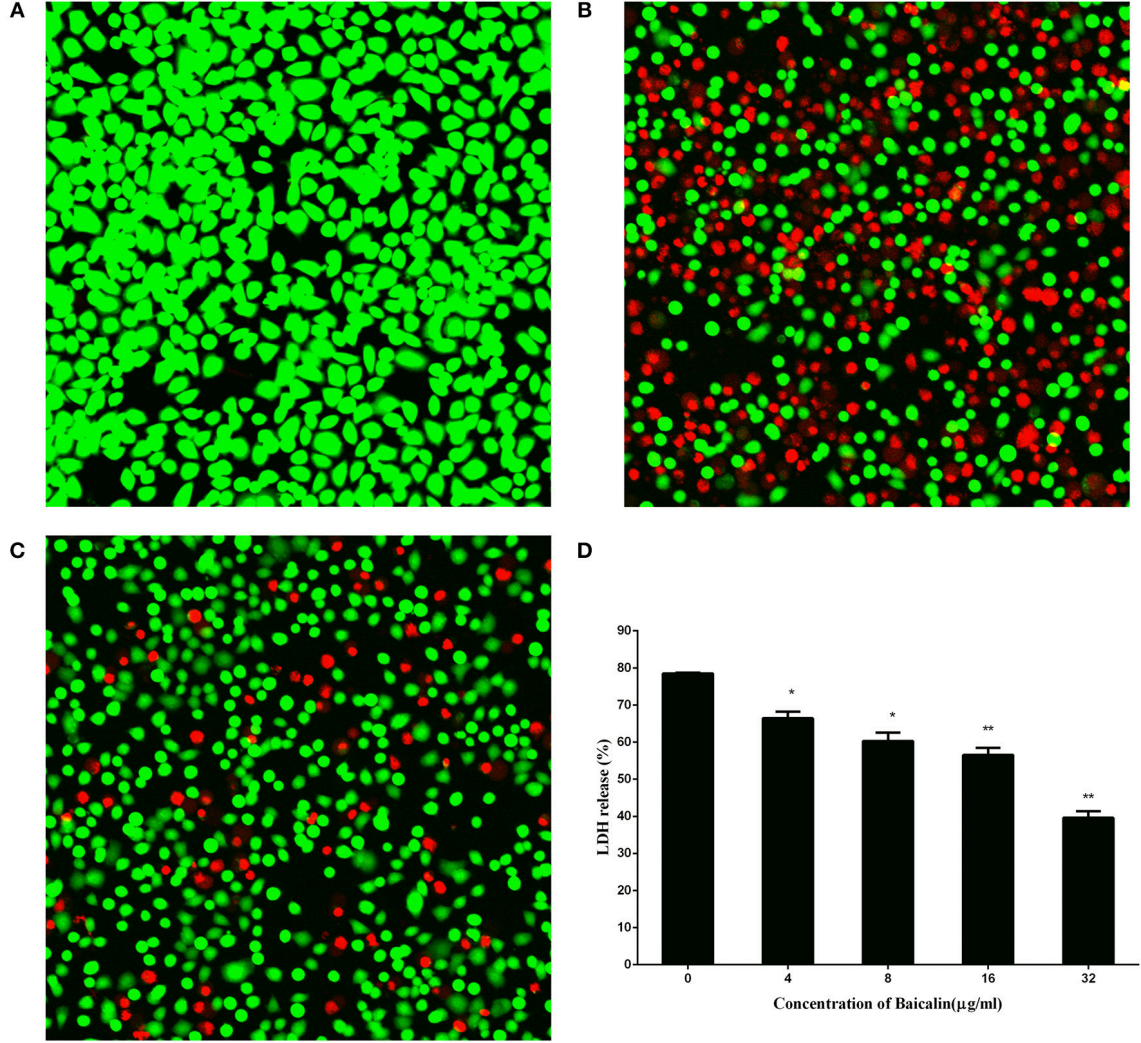
The results of the live (green)/dead (red) assay and LDH release by A549 cells after incubation with *S. aureus* and various concentrations of baicalin. **(A)** Cells treated with DMEM only, **(B)** Cells co-cultured with *S. aureus* without baicalin treatment, **(C)** Cells co-cultured with *S. aureus* and 32 μg/ml of baicalin, **(D)** The LDH release by A549 cells after incubation with *S. aureus* and various concentrations of baicalin. The scale bars are 70, 20, and 30 μm for **(A–C)**, respectively. The data are presented as mean values and standard deviations of three independent experiments. *p < 0.05 and **p < 0.01.

Methicillin-resistant *S. aureus* (MRSA) has become one of the leading pathogens of nosocomial pneumonia as a result of an increase in the prevalence of *S.aureus* infections (Jiang et al., 2017). A549 cells, a human alveolar epithelial cell model, are widely used in the evaluation of pulmonary disease. Here, to assess the preventive effect of baicalin on *S. aureus* pneumonia, human alveolar epithelial A549 cells were subjected to live/dead and LDH assays. As shown in Figure 3A, the cells cultured in DMEM without *S. aureus* manifested a complete presence of green fluorophores, indicating that the cells were alive. Conversely, as depicted in Figure 3B, cells treated with DMEM containing *S. aureus* showed a widespread presence of red fluorophores, proving that *S. aureus* caused major damage to A549 cells. Nevertheless, the intensity of red fluorophores significantly diminished after A549 cells were co-cultured with *S. aureus* and 32 μg/ml baicalin. These data suggested that baicalin effectively protects A549 cells from damage by *S. aureus* infection (Figure 3C).

LDH is a vital indicator of cell membrane integrity and has been extensively used as an indicator of cell viability. The results of the LDH release assay (Figure 3D) suggested that baicalin exerted a protective effect on A549 cells in a dose-dependent manner from a concentration of 4 μg/ml to 32 μg/ml, thus providing more evidence that baicalin protects A549 cells from *S. aureus*.

### Baicalin weakened the adhesion of *S. aureus* to A549 cells

Adhesion to host tissue is an essential step for gram-positive pathogens, including *S. aureus*, for successful infection, (Cascioferro et al., 2014). Compounds that block the pathogen's adherence to host tissue can serve as potential alternatives or adjunctive therapeutics compared to conventional antibiotics for the control of infection (Cascioferro et al., 2014).

**Figure 4:**
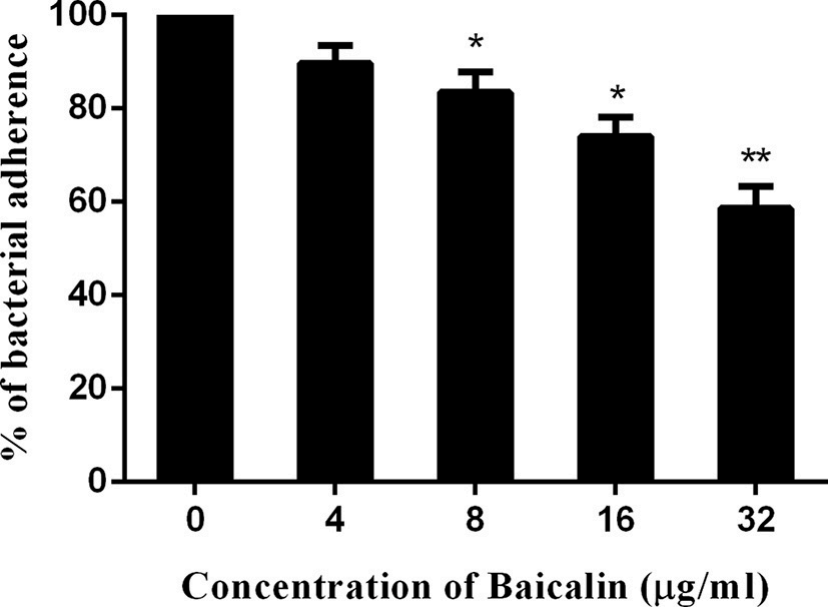
Baicalin weakens the adhesion of *S. aureus* to A549 cells. The data are shown as mean values and standard deviation of three repetitions; *p < 0.05 and **p < 0.01. The results are presented as the proportion of colony counts adhered to A549 cells relative to the original inocula.

Some studies have revealed that SrtB plays a role in the adhesion of *S. aureus* to host tissue (Jonsson et al., 2003). Based on this observation, an adhesion assay was performed and revealed that baicalin weakened the adhesion of *S. aureus* to A549 cells in a dose-dependent manner under the given conditions (Figure 4).

### Baicalin reduced the inflammatory response caused by *S. aureus*

*S. aureus* pneumonia has become one of the most prevalent diseases in hospital- and community-acquired pneumonia. The inflammation caused by the *S. aureus* SrtB knockout strain was lower than that of wild type *S. aureus* (Jonsson et al., 2003), suggesting SrtB plays a role in the inflammation caused by *S. aureus*.

To explore the latent mitigating role of baicalin on the inflammation generated by *S. aureus*, an enzyme-linked immune-sorbent assay was carried out to determine the cytokine in the culture media of J774 mouse macrophage cells treated with *S. aureus*.

**Figure 5:**
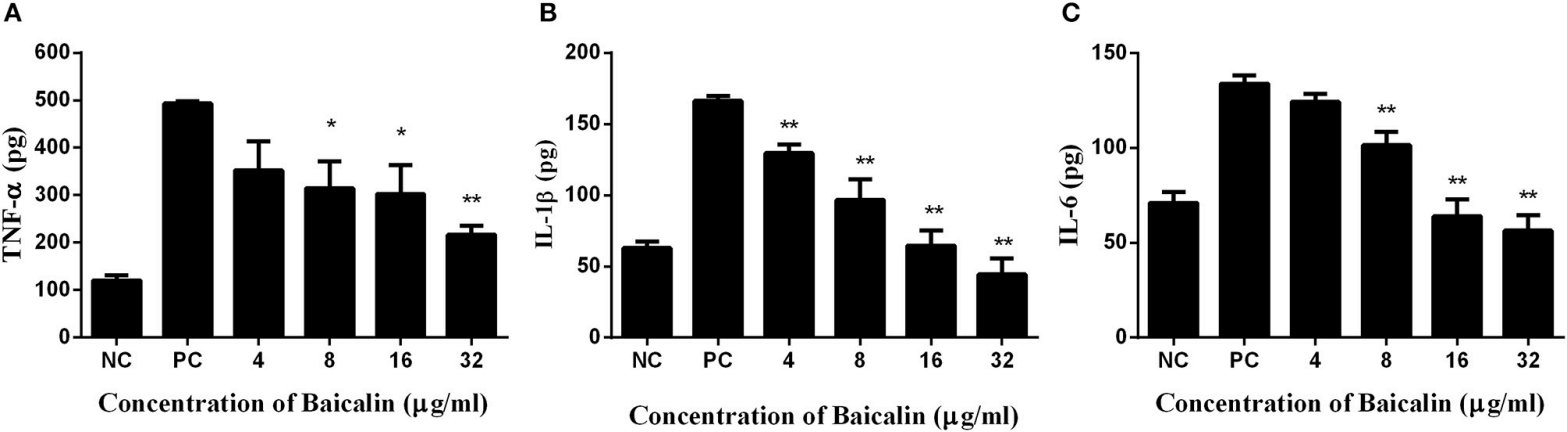
The inflammatory response of J774 cells after treatment with *S. aureus* and various concentrations of baicalin. **(A)** The concentration of TNF-α in the culture medium. **(B)** The concentration of IL-1β in the culture medium. **(C)** The concentration of IL-6 in the culture medium. The data are presented as mean ± SEM (*n* = 3); *p < 0.05 and **p < 0.01.

Mouse macrophage J774 cells have been popular as a cell model for the evaluation of inflammatory factors. As shown in Figure 5, concentrations of TNF-α, IL-1β, and IL-6 were significantly lower in the culture supernatant of J774 cells treated with *S. aureus* plus different concentrations of baicalin, in comparison with the supernatant of the cells treated with *S. aureus* alone. As for TNF-α, a statistically significant difference was detected when the concentration of baicalin reached 8 μg/ml, with a value of 314.866 pg for the cytokine as compared to 493.367 pg in the positive control group. When the concentration of baicalin was 32 μg/ml the difference became even more drastic, i.e., 216.769 pg of TNF-α compared with 493.367 pg in the positive control group (Figure 5A). Similarly, the levels of IL-1β decreased with the increasing concentrations of baicalin, where a statistically significant difference was detected for the baicalin concentration as low as 4 μg/ml (Figure 5B). When the concentration of baicalin was 8 μg/ml a statistically significant difference was detected in the level of IL-6 which was 101.464 pg compared with 133.908 pg in the positive control group (Figure 5C). All these data indicated that baicalin can significantly reduce the inflammatory response elicited by *S. aureus*.

### Prediction of the binding model between SrtB and baicalin

**Figure 6:**
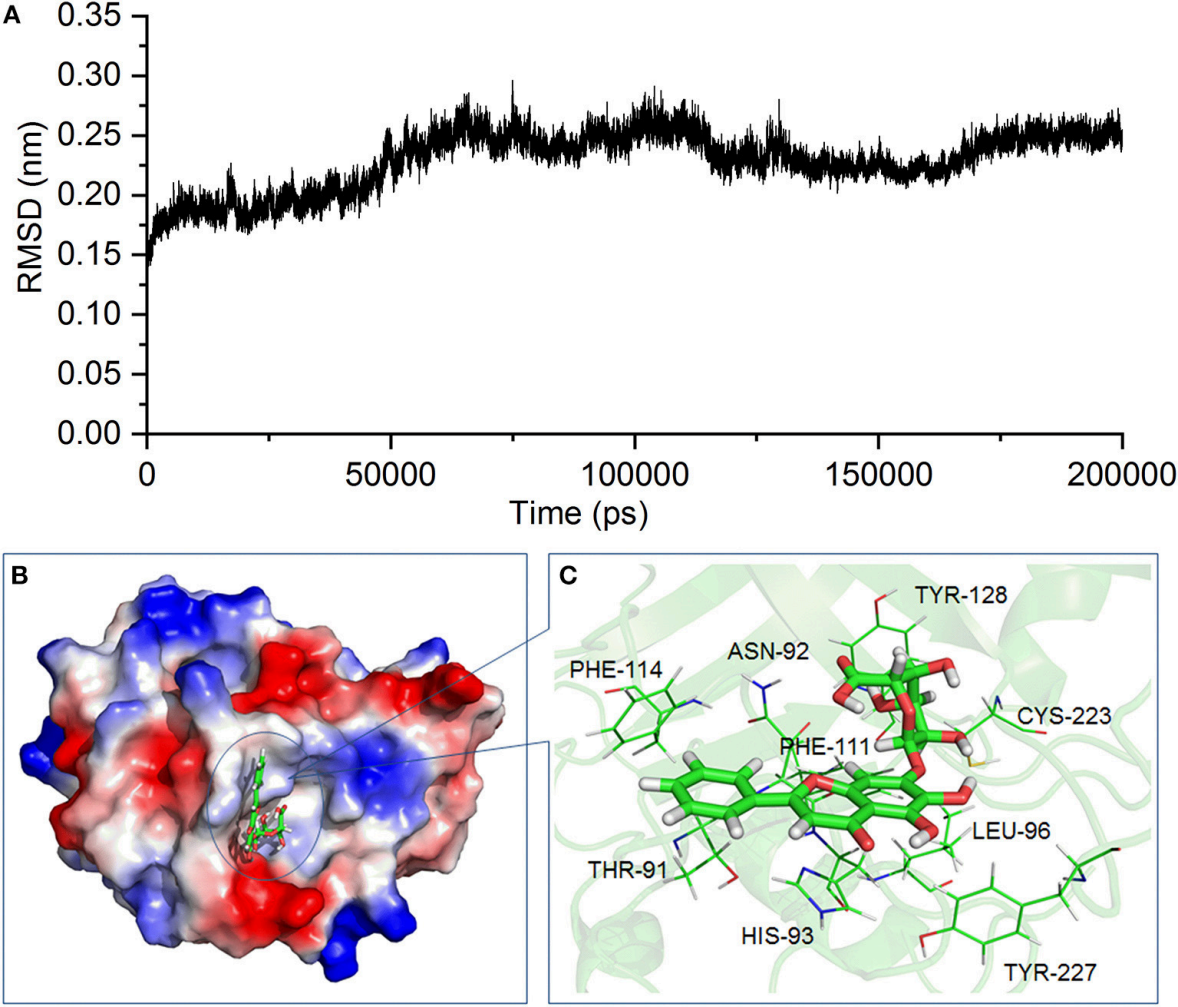
The 3D structure of the SrtB–baicalin complex determined by molecular modeling. **(A)** The root mean square deviation (RMSD) of the SrtB–baicalin complex. **(B)** The binding model of baicalin with SrtB. **(C)** The specific details on the residues that are bound to baicalin.

According to the inhibitory effect of baicalin on the activity of SrtB, we assume that baicalin may directly interact with SrtB. Molecular modeling and molecular docking analysis of the SrtB–baicalin complex were carried out to assess the interaction of the protein and ligand. The 3D structure of the SrtB–baicalin complex was confirmed during the 200–ns simulation. As shown in Figure 6A, the SrtB–baicalin complex reached equilibrium at 60 ns according to root mean square deviations of the protein, suggesting that the structure of the SrtB–baicalin complex studied in the following assays was reliable.

**Figure 7:**
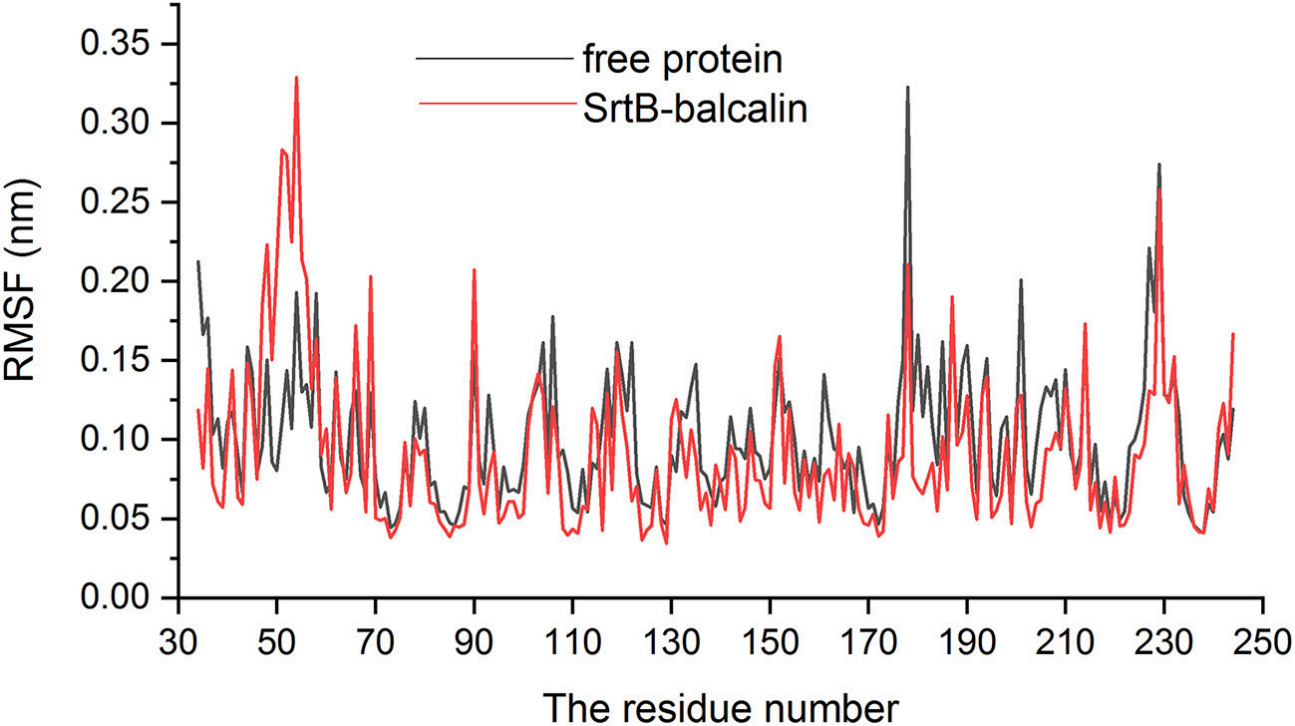
The RMSF of residues in free protein (black line) and SrtB–baicalin complex (red line).

As presented in Figure 6B, baicalin binds to the catalytic reaction region (active pocket) of SrtB, and the Van der Waals force and hydrogen bonds contribute to the binding process. Of note, during the binding process, the conformation of baicalin was similar to “L.” More specifically, residues Thr^91^, Phe^114^, and His^93^ were close to the conjugate ring group on the long side of “L,” and engaged in a strong interaction with baicalin judging by data in Figure 6C. Additionally, residues Tyr^128^ and Cys^223^ are closer to the short side of the “L” than to other residues, indicating that the short side of baicalin was fixed by Tyr^128^ and Cys^223^. The conjugate ring on the corner of baicalin was found to be in much closer proximity to Phe^111^, Leu^96^, Asn^92^, and Tyr^227^; thus, a strong interaction emerged between them. Next, we analyzed the root mean square fluctuations (RMSFs) to assess the flexibility changes in the residues bound to baicalin. As illustrated in Figure 7, the RMSF values of Thr^91^, Asn^92^, His^93^, Leu^96^, Phe^111^, Phe^114^, Tyr^128^, Cys^223^, and Tyr^227^ were lower in the SrtB–baicalin complex than in the free protein, with all the values being < 0.05 nm. This result suggested that flexibility of the peptide chain containing these residues was weakened, and confirmed that these residues were in the binding sites of SrtB and baicalin. The residues became restrained (the peptide chain became more rigid) after binding with baicalin, thus causing the observed loss of flexibility.

### Confirmation of the binding sites for baicalin in SrtB

For further exploration of the contributions of amino acid residues at the site of binding between baicalin and SrtB, we employed the Molecular Mechanics Poisson–Boltzmann Surface Area method to calculate the binding free energy of baicalin with SrtB.

**Figure 8:**
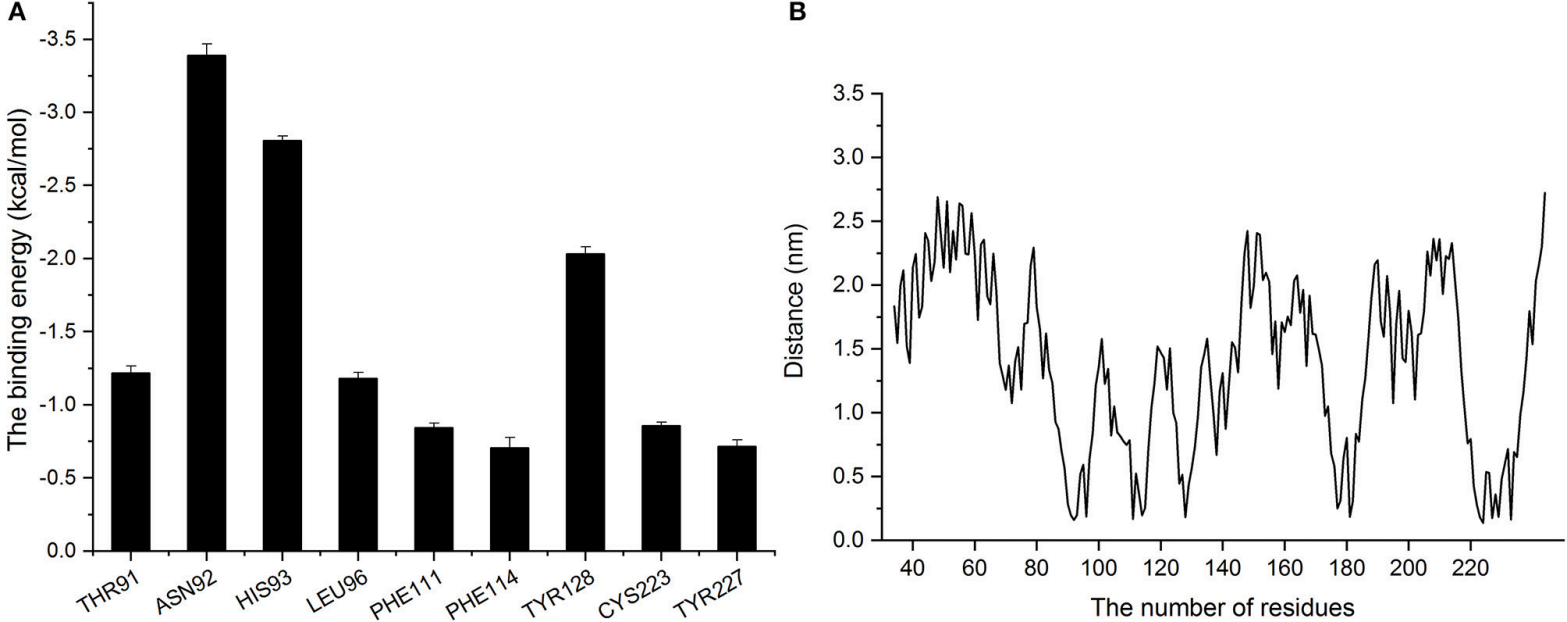
Analysis of binding energy and the distance between baicalin and amino acid residues of SrtB. **(A)** The binding energy of residues that bound to baicalin. **(B)** The distance between residues of SrtB and baicalin during the molecular dynamics simulation.

As presented in Figure 8A, residues Asn^92^ and Leu^96^ showed greater binding energy with baicalin according, with Δ*E*_*total*_ values of −3.39 and −1.18 kcal/mol, respectively, confirming that Asn^92^ and His^93^ greatly contribute when the center (the corner part of the “L”) of baicalin is bound to the active region of SrtB. In addition, residues His^93^ and Thr^91^ also contribute to the binding free energy, with Δ*E*_*total*_ values of −2.8 and −1.22 kcal/mol, suggesting that baicalin was stabilized by these two residues via the formation of a strong interaction at the long side of baicalin. The Δ*E*_*total*_ value of Tyr^128^ was −2.034 kcal/mol, clearly indicating that Tyr^128^ was the most important residue for binding of the short side of baicalin to the active pocket of SrtB. The other residues (Phe^111^, Phe^114^, Cys^223^, and Tyr^227^) had Δ*E*_*total*_ values less than −1.00 kcal/mol, indicating that these residues were less important for anchoring baicalin to SrtB.

The distances between each residue and baicalin were calculated when the complex reached an equilibrium. On the basis of the results in Figure 8A, we found that the higher the binding energy, the shorter the distance between baicalin and SrtB was, and the distances were < 0.25 nm (Figure 8B) Therefore, according to these data, residues Thr^91^, Asn^92^, His^93^, Leu^96^, Phe^111^, Phe^114^, Tyr^128^, Cys^223^, and Tyr^227^ likely actively participate in the binding of baicalin to SrtB. Specifically, we show that Asn^92^ helps to fix the center of baicalin (the corner part of the “L”), His^93^ is involved in anchoring the long side of baicalin, and Tyr^128^ fixes the short side of baicalin to SrtB.

For confirmation, a molecular dynamics simulation assay was performed to analyze the binding of N92A and Y128A mutants to baicalin. The molecular mechanics Poisson–Boltzmann surface area analysis was used to calculate the binding free energies between the mutants and the ligand. Simultaneously, the fluorescence spectroscopy quenching (Qiu et al., 2012; Wang et al., 2015) method was carried out to determine the binding constants (*K*_*A*_) of the complexes mentioned above.

**Figure 9:**
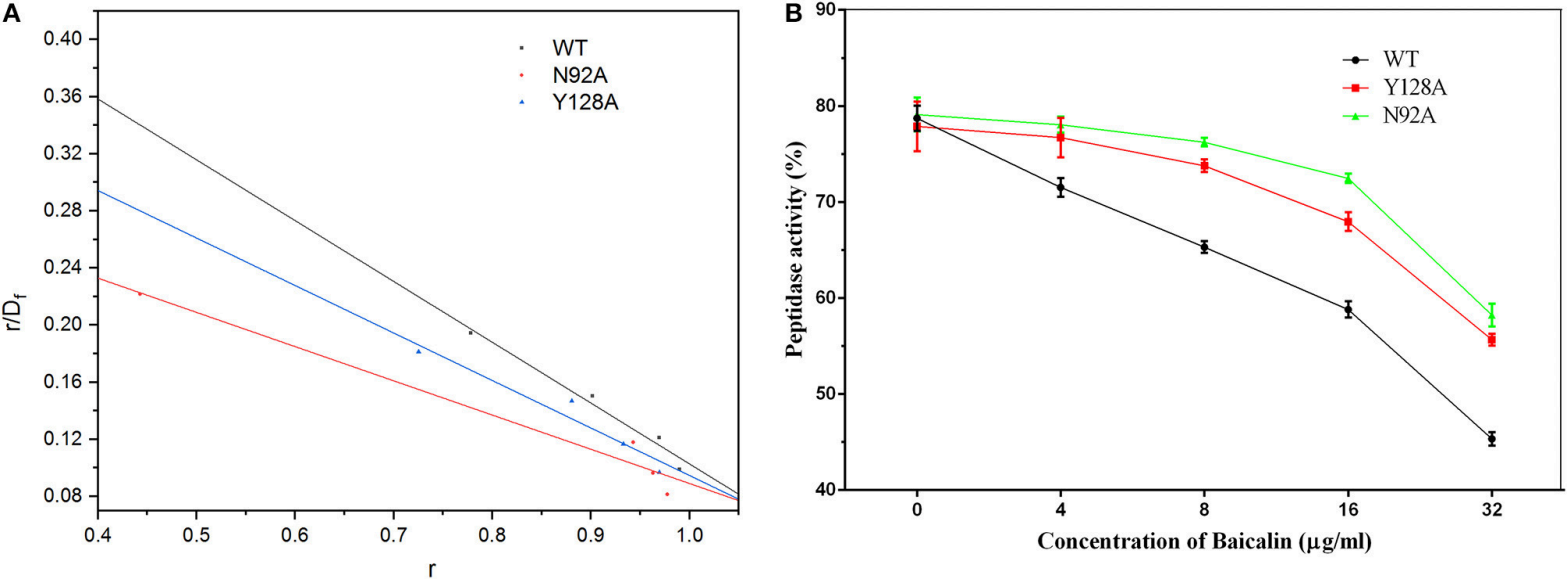
The fitted linear based on the fluorescence quenching assay and the inhibitory effect of baicalin on SrtB and its mutants. **(A)** The fitted linear of r/D_f_ vs. r for the binding of baicalin to WT SrtB and SrtB mutants N92A, and Y128A. **(B)** The inhibitory effects of baicalin on SrtB and on the mutants. The data are shown as mean values and standard deviation of three repetitions.

During the fluorescence spectroscopy quenching assay, the fluorophore was the protein and the quencher was baicalin. The Scatchard equation was derived to formulate the assay as follows: r/D_f_ = nK–rK. In the equation, K represents the binding constant, D_f_ is the free concentration of baicalin, r is the ligand amount of substance per mole of protein binding (r ≈ΔF/F0), and n is the number of binding sites. The concentration of baicalin in the interaction system was much higher than the concentration of protein. Accordingly, D_f_ was replaced with the total baicalin concentration. Figure 9A shows the fitted linear values, and the resulting *K*_*A*_values are given in Table 2.

**Table 2:** The binding energies and the binding constants (*K*_*A*_) between proteins and baicalin.

	**WT-SrtB**	**N92A**	**Y128A**
ΔG_bind_(kcal/mol)	−39.8 ± 2.8	−28.8 ± 2.4	−31.0 ± 3.4
*K_*A*_* (1 × 10^4^) L·mol^−1^	19.0 ± 1.26	10.7 ± 1.35	14.8 ± 1.43

The results presented in Table 2 reveal that the binding energies of the SrtB mutants toward baicalin were weaker than those of WT SrtB with baicalin and could be ranked in the following order: WT > Y128A > N92A. Moreover, according to the results of the fluorescence spectroscopy quenching assay, the binding constants (*K*_*A*_) of the ligand with the mutant proteins were lower and could be ranked in the following order: WT > Y128A > N92A, revealing high consistency with the theoretical calculation results presented in Table 2. Therefore, it is convincing that the binding model of SrtB with baicalin was reliably obtained by the molecular dynamics simulation.

We evaluated the inhibitory effect of baicalin on the mutants as depicted in Figure 9B. The mutants turned out to have bioactivity comparable to that of WT SrtB. The inhibitory effect of baicalin on the SrtB mutants was clearly weaker than that on WT SrtB and the tendency conformed to the binding energy and binding constant, further confirming the results of the molecular dynamics simulation.

In summary, during binding to SrtB, baicalin is located in the catalytic region of SrtB, and residues of Asn^92^ and Tyr^128^ play a significant role in the binding process. Additionally, baicalin occupies the active center of SrtB, thereby causing a loss of enzymatic activity.

## Discussion

The emergence of multidrug-resistant strains of *S. aureus* has resulted in extreme challenges to the treatment of this pathogen (Chambers and Deleo, 2009). Thus, the development of new and effective therapeutic strategies is of paramount importance. Anti-virulence strategies that involve virulence factors as drug targets show promise as alternative or adjunctive modalities to combat the infections with pathogenic bacteria (Rasko and Sperandio, 2010).

SrtB is a virulence factor found in *S. aureus* and has been reported to play a crucial part in the course of infection. Nevertheless, reports of SrtB inhibitors are few. Here, we studied SrtB as a therapeutic target and identified its inhibitor. Baicalin exerts no growth pressure on *S. aureus* but inhibits SrtB activity *in vitro*. The half-maximal inhibitory concentration (IC_50_) was 25.86 μg/ml. Live/dead and LDH assays suggest that baicalin protects human alveolar epithelial cells from injury caused by *S. aureus*. An adherence assay indicates that baicalin attenuates the adhesion of *S. aureus* to these cells. ELISAs confirmed that baicalin reduces inflammatory activities of mouse macrophage cell lines that are associated with *S. aureus* infection.

Moreover, we verified the binding model and identified key residues involved in the binding process. Via a 200 ns molecular dynamics simulation, we found that baicalin is fixed in the active center of SrtB, and the residues Asn^92^ and Tyr^128^ play critical roles during the binding process. We also analyzed the flexibility changes in residues bound to baicalin by an RMSF assay and found that the flexibility of those residues is lower in the SrtB–baicalin complex than in free SrtB, thus confirming the results of the molecular dynamics simulation. For further validation, we carried out site-directed mutagenesis and fluorescence spectroscopy quenching assays and revealed that the binding constant (*K*_*A*_) of WT SrtB for baicalin is stronger than that of SrtB mutants. These data are well in agreement with the binding free energies between proteins and baicalin according to theoretical calculations. Additionally, the inhibitory effects of baicalin on SrtB diminished after site-directed mutagenesis, indicating that the results of the molecular dynamics simulation were reliable. Taken together, these results indicate that baicalin occupies the active center of SrtB and blocks the binding to the substrate, causing a loss of SrtB activity.

In conclusion, baicalin inhibits SrtB activity significantly *in vitro*, and exerts no growth pressure on *S. aureus*. Moreover, baicalin protects human alveolar epithelial cells from *S. aureus*, weakens the adhesion of *S. aureus* to human alveolar epithelial cells, and attenuates the inflammatory response of mouse macrophages (cell line J774) to *S. aureus*. Furthermore, baicalin directly binds to the active pocket of SrtB to block the binding of SrtB with its substrate, causing a decrease in the activity of SrtB.

## Author contributions

XN and JW conceived and designed the experiments. GW and YG performed the experiments. XN, HW, and YG contributed reagents, materials, analysis tools. XN, JW, and GW wrote the paper.

### Conflict of interest statement

The authors declare that the research was conducted in the absence of any commercial or financial relationships that could be construed as a potential conflict of interest.
