# Supplementary material for: Baicalin Weakens Staphylococcus aureus Pathogenicity by Targeting Sortase B
Source: Front Cell Infect Microbiol. 2018 Nov 30;8:418. doi: 10.3389/fcimb.2018.00418 (PMC6284026; doi:10.3389/fcimb.2018.00418)
Supplement: Supplementary file 1 [file Data_Sheet_1.pdf]

Baicalin Weakens *Staphylococcus aureus* Pathogenicity by Targeting Sortase B

Guizhen Wang[1#], Yawen Gao[1#], Hongsu Wang[1] Xiaodi Niu[1*] & Jianfeng Wang[2*]

[1] College of Food Science and Engineering, Jilin University, Changchun, China
[2] Key Laboratory of Zoonosis, Ministry of Education, College of Veterinary Medicine, Jilin University, Changchun, China

[*] Correspondence and requests for materials should be addressed to X.D.N. (niuxd@jlu.edu.cn) or J.F.W. (wjf927@jlu.edu.cn)

[#]These authors contributed equally to this work.

Method and computation

Molecular docking calculation

The crystal structure of SrtB was obtained from the Protein Data Bank (PDB), and PDB code 1NG5 was used as the initial coordinates for the molecular docking calculations using AutoDock 4.0 (Morris et al., 1996;Hu et al., 2010). The Lamarckian genetic algorithm (LGA) was applied for the docking calculations. All of the torsional bonds of the drug were free to rotate, while SrtB was held rigid. The polar hydrogen atoms were added for SrtB using the AutoDock tools, and the Kollman united atom partial charges (Morris et al., 1996) were assigned. A total of 150 independent runs were carried out with a maximum of 25,000,000 energy evaluations and a population size of 300. A grid box of dimensions (40×40×40) with a spacing of 1 Å was created and centred on the mass centre of the ligand. Energy grid maps for all possible ligand atom types were generated by using Autogrid 4 before performing docking.

Molecular dynamics simulation

All of the simulations and analyses of the trajectories were performed with Gromacs 4.5.1 software (Hess et al., 2008) using the Amber99sb force field and TIP3P water model (Jorgensen et al., 1983). The SrtB-baicalin system was initially energy-relaxed with 2000 steps of the steepest-descent energy minimization and 2000 subsequent steps of conjugate-gradient energy minimization. The system was then equilibrated by a 500 ps molecular dynamic run with positional restraints on both the protein and ligand to allow relaxation of the solvent molecules. The first equilibration run was followed by a 100 ns MD run without position restraints on the solute. The first 20 ns of the trajectory were not used in the subsequent analysis for minimization of convergence artefacts. Equilibration of the trajectory was checked by monitoring the equilibration of quantities, such as the root-mean-square deviation (rmsd) with respect to the initial structure, internal protein energy and fluctuations calculated for different time intervals. The electrostatic term was described with the particle mesh Ewald algorithm. The LINCS (Ryckaert et al., 1977) algorithm was used to constrain all of the bond lengths. For the water molecules, the SETTLE algorithm (Ryckaert et al., 1977) was used. The dielectric permittivity ε was set as 1, and a time step of 2 fs was used. All atoms were given an initial velocity as determine from the Maxwell distribution at the desired initial temperature of 300 K. The density of the system was adjusted during the first equilibration runs under NPT conditions by weak coupling to a bath of constant pressure (P_0 = 1 bar, coupling time τ_P = 0.5 ps) (Berendsen et al., 1984). In all of the simulations the temperature was maintained close to the intended values by weak coupling to an external temperature bath with a coupling constant of 0.1 ps. The proteins and rest of the system were coupled separately to the temperature bath. The structural cluster analysis was carried out using the method described by Daura and co-workers with a cutoff of 0.25 nm (Berendsen et al., 1984).

The parameters of baicalin was estimated with the antechamber program (Wang et al., 2006) and the RESP partial atomic charges from the Amber suite (Jakalian et al.,

2002). Analysis of the trajectories was performed by using the VMD, PyMOL and Gromacs analysis tools.

Calculation of the binding free energy

In this work, the binding free energies were calculated using the MM-PBSA approach (Punkvang et al., 2010;Schaffnerbarbero et al., 2010) supplied with the Amber 10 package. We chose a total number of 100 snapshots evenly from the last 70 ns on the MD trajectory with an interval of 10 ps. The MM-PBSA method can be conceptually summarized as one

$$\Delta G_{bind}=\Delta G_{complex}-[\Delta G_{protein}+\Delta G_{lig}] \quad 1$$

$$\Delta G_{bind}=\Delta H-T\Delta S \quad 2$$

which the ΔH of the system is composed of the enthalpy changes in the gas phase upon complex formation (ΔE_{MM}) and the solvated free energy contribution (ΔG_{sol}), while $-T\Delta S$ refers to the entropy contribution to the binding. Eq. 2 can then be approximated as shown in Eq. 3:

$$\Delta G_{bind}=\Delta E_{MM}+\Delta G_{sol}-T\Delta S \quad 3$$

where ΔE_{MM} is the sum of the van der Waals (ΔE_{vdw}) and electrostatic (ΔE_{ele}) interaction energies.

$$\Delta E_{MM}=\Delta E_{vdw}+\Delta E_{ele} \quad 4$$

In addition, ΔG_{sol}, which denotes the solvation free energy, can be computed as the sum of an electrostatic component ($\Delta G_{ele,sol}$) and a nonpolar component ($\Delta G_{nonpolar,sol}$), as shown in Eq. 5:

$$\Delta G_{sol}=\Delta G_{ele,sol}+\Delta G_{nonpolar,sol} \quad 5$$

The interactions between baicalin and each residue in the binding site of SrtB with baicalin were analysed using the MM-PBSA decomposition process applied in the MM-PBSA module in Amber 10. The binding interaction of each ligand-residue pair includes three terms, namely, the Van der Waals contribution (ΔE_{vdw}), electrostatic contribution (ΔE_{ele}) and solvation contribution (ΔE_{sol}). All of the energy components were calculated using the same snapshots as the free energy calculation.

Table S1. Primers used in this study

Name	Oligonucleotide (5'-3')*
$SrtB_{\Delta30}$-F	CGCGGATCCGAAGACAAGCAAGAACGCGC
$SrtB_{\Delta30}$-R	CCGCTCGAGTTAACTTACCTTAATTATTTTTGCGACAAC
$SrtB_{\Delta30}$-N92F	GTACTACAAGGTAAGACAGCGCACGATTATTTAAATTTAG
$SrtB_{\Delta30}$-N92R	CTAAATTTAAATAATCGTGCGCTGTCTTACCTTGTAGTAC
$SrtB_{\Delta30}$-Y128F	CATAATACTATTTTAGCGGGGCACCATGTCGG
$SrtB_{\Delta30}$-Y128R	CCGACATGGTGCCCCGCTAAAATAGTATTATG
$SrtB_{\Delta30}$-Y224F	GACTTTATCAACGTGCGCGGATGCATATAGTGAAAC
$SrtB_{\Delta30}$-Y224R	GTTTCACTATATGCATCCGCGCACGTTGATAAAGTC
$SrtB_{\Delta30}$-Y233F	GTGAAACAACGAAAGCGATTGTTGTTGTCGC
$SrtB_{\Delta30}$-Y233R	GCGACAACAACAATCGCTTTCGTTGTTTCAC

*The underlined basic group represents restriction endonuclease recognition sites or mutated codons.

Reference

Berendsen, H.J.C., Postma, J.P.M., Gunsteren, W.F.V., Dinola, A., and Haak, J.R. (1984). Molecular dynamics with coupling to an external bath. *Journal of Chemical Physics* 81**,** 3684-3690.

Hess, B., Kutzner, C., Van, D.S.D., and Lindahl, E. (2008). GROMACS 4: Algorithms for Highly Efficient, Load-Balanced, and Scalable Molecular Simulation. *Journal of Chemical Theory & Computation* 4**,** 435.

Hu, R., Barbault, F., Maurel, F., Delamar, M., and Zhang, R. (2010). Molecular Dynamics Simulations of 2-Amino-6-arylsulphonylbenzonitriles Analogues as HIV Inhibitors: Interaction Modes and Binding Free Energies. *Chemical Biology & Drug Design* 76**,** 518–526.

Jakalian, A., Jack, D.B., and Bayly, C.I. (2002). Fast, efficient generation of high-quality atomic charges. AM1-BCC model: II. Parameterization and validation. *Journal of Computational Chemistry* 23**,** 1623-1641.

Jorgensen, W.L., Chandrasekhar, J., Madura, J.D., Impey, R.W., and Klein, M.L. (1983). Comparison of simple potential functions for simulating liquid water. *Journal of Chemical Physics* 79**,** 926-935.

Morris, G.M., Goodsell, D.S., Huey, R., and Olson, A.J. (1996). Distributed automated docking of flexible ligands to proteins: Parallel applications of AutoDock 2.4. *Journal of Computer-Aided Molecular Design* 10**,** 293-304.

Punkvang, A., Saparpakorn, P., Hannongbua, S., Wolschann, P., Beyer, A., and Pungpo, P. (2010). Investigating the structural basis of arylamides to improve potency against M. tuberculosis strain through molecular dynamics simulations. *European Journal of Medicinal Chemistry* 45**,** 5585-5593.

Ryckaert, J.P., Ciccotti, G., and Berendsen, H.J.C. (1977). Numerical integration of the cartesian equations of motion of a system with constraints: molecular dynamics of n -alkanes. *Journal of Computational Physics* 23**,** 327-341.

Schaffnerbarbero, C., Gilredondo, R., Ruizavila, L.B., Huecas, S., Läppchen, T., Den, B.T., Diaz, J.F., Morreale, A., and Andreu, J.M. (2010). Insights into nucleotide recognition by cell division protein FtsZ from a mant-GTP competition assay and molecular dynamics. *Biochemistry* 49**,** 10458-10472.

Wang, J., Wang, W., Kollman, P.A., and Case, D.A. (2006). Automatic atom type and bond type perception in molecular mechanical calculations. *Journal of Molecular Graphics and Modelling* 25**,** 247-260.
